# Impact of specialist ataxia centres on health service resource utilisation and costs across Europe: cross-sectional survey

**DOI:** 10.1186/s13023-023-02971-4

**Published:** 2023-12-07

**Authors:** Stephen Morris, Julie Vallortigara, Julie Greenfield, Barry Hunt, Deborah Hoffman, Carola Reinhard, Holm Graessner, Antonio Federico, Vinciane Quoidbach, Paola Giunti

**Affiliations:** 1 Primary Care Unit, Department of Public Health and Primary Care, University of Cambridge, East Forvie Building, Forvie Site, Robinson Way, Cambridge, CB2 0SR UK; 2 Ataxia Centre, Queen Square Institute of Neurology, Department of Molecular and Movement Neurosciences, University College London, Queen Square House, London, WC1N 3BG UK; 3 Ataxia UK, London, UK; 4 Takeda Pharmaceuticals, Cambridge, MA USA; 5 Centre for Rare Diseases and Institute of Medical Genetics and Applied Genomics, University Hospital Tübingen, Tübingen, Germany; 6 Department of Medicine, Surgery and Neurosciences, Medical School, University of Siena, Siena, Italy; 7 European Academy of Neurology, Vienna, Austria; 8 European Brain Council, Brussels, Belgium

**Keywords:** Health service use, Costs, Ataxia, Specialist centre

## Abstract

**Background:**

Little is known about the costs of treating ataxia and whether treatment at a specialist ataxia centre affects the cost of care. The aim of this study was to investigate whether patients who attended specialist ataxia centres in three European countries reported differences in their health care use and costs compared with patients who did not attend a specialist ataxia centre. We compared mean resource use and health service costs per patient affected by ataxia in the United Kingdom, Italy and Germany over a 12-month period. Data were obtained from a survey distributed to people with ataxia in the three countries. We compared mean resource use for each contact type and costs, stratifying patients by whether they were currently attending a specialist ataxia centre or had never attended one.

**Results:**

Responses were received from 181 patients from the United Kingdom, 96 from Italy and 43 from Germany. Differences in the numbers of contacts for most types of health service use between the specialist ataxia centre and non-specialist ataxia centre groups were non-significant. In the United Kingdom the mean total cost per patient was €2209 for non-specialist ataxia centre patients and €1813 for specialist ataxia centre patients (*P* = 0.59). In Italy these figures were €2126 and €1971, respectively (*P* = 0.84). In Germany they were €2431 and €4087, respectively (*P* = 0.19). Inpatient stays made the largest contribution to total costs.

**Conclusions:**

Within each country, resource use and costs were broadly similar for specialist ataxia centre and non-specialist ataxia centre groups. There were differences between countries in terms of health care contacts and costs.

**Supplementary Information:**

The online version contains supplementary material available at 10.1186/s13023-023-02971-4.

## Introduction

The ataxias are a group of complex rare neurological disorders, for which more than one hundred genetic causes have been identified [1]. Patients living with ataxia can experience a range of symptoms resulting from the damage to the cerebellum or its connections. Features of ataxia include gait instability, variably associated with dysarthria, double vision, tremor, peripheral neuropathy, pyramidal and extra pyramidal symptoms and cognitive impairment [2, 3]. Global epidemiological studies have estimated an overall ataxia prevalence rate of 26 cases per 100,000 in children, and for hereditary cerebellar ataxia a prevalence rate of 2.7 to 3.3 cases per 100,000 [4]. Friedreich’s ataxia is the most common inherited ataxia, with an estimated prevalence of 3.4 cases per 100,000 individuals [5]. Patients with ataxia often have significant needs that require a complex package of health and social care, involving input with numerous health professionals, including neurologists, general practitioners, ophthalmologists, urologists, ear nose and throat specialists, gastroenterologists, cardiologists and other specialists addressing specific symptomatic treatments. For holistic treatment of ataxia, patients are also often referred to physiotherapists, speech and language therapists and occupational therapists. Given its rarity, and a lack of awareness and understanding of ataxia among health professionals, accessing suitable care for this condition can be challenging.

Many European countries have implemented health policies with the aim of improving care for people with rare conditions. Due to the specific expertise often required to manage rare conditions such as ataxia, expert or specialist centres are often highlighted as a principal mechanism of effective treatment. Specialist centres aim to “provide comprehensive, integrated, multidisciplinary care for patients, as well as information and support for family members. They aim to incorporate networks of all relevant medical disciplines within the core team. They have effective links with national networks of testing laboratories and other care centres at a national and international level.” [6] There are 84 expert centres in rare neurological diseases across Europe that are part of the European Reference Network for Rare Neurological Diseases (ERN-RND) [7]. Specialist ataxia centres (SAC) can provide the necessary coordinated care and therefore address the specific and varied needs of ataxia patients [8].

We have reported previously on the patient pathways for the management of ataxia in three European countries [9]. However, it is not known if treatment at a SAC is associated with higher or lower use of health care resources and costs. The aim of this study was to investigate whether patients who attended a SAC in three European countries reported differences in their health care utilisation and costs compared with patients who did not attend a SAC. This research is part of the Value of Treatment (VOT) project supported by The European Brain Council (EBC), to highlight the burden of neurological disorders and improve the care and outcomes of people living with ataxia and other neurological disorders across Europe [10].

## Methods

### Survey instrument

In the UK at the time the survey was conducted there were two SACs (in London and Sheffield); in German there were nine (in Lunbeck, Munchen, Tubingen, Bonn, Essen, Aachen, Berlin, Dusseldorf and Magdeburg); and, in Italy there were eleven (in Florence, Milan, Messina, Naples, Rome (2 centres), Siena, Turin, Pisa, Genova and Bologna). We conducted a survey of patients and families affected by ataxia in these three countries, collecting data on their use of health services and whether or not they attended a SAC for treatment. We also collected data on socio-demographic characteristics and other factors relating to the diagnosis and management of the condition. The survey questionnaire was developed with input from patient group representatives, a specialist ataxia neurologist, a specialist ataxia nurse, a health economist and representatives of pharmaceutical companies involved in ataxia research. The questionnaire mainly contained close-ended questions with defined response categories. Participants were provided with an information sheet about the survey, which included the purpose of the study, the organisations involved, and assurances around anonymity and aggregation of data for reporting purposes. The first part of the questionnaire explained the context of the study and the medical terms used. Participants were then asked a series of questions about their socio-demographic characteristics, including the type of ataxia they were affected by. The next section of the survey asked about experiences of diagnosis. This was followed by a series of questions about health service contacts of different types, and about whether or not the person affected by ataxia attended a SAC for some or all of their treatments, and if so, their experiences of this. Participants were then asked a series of questions about the treatment they received and their satisfaction with this. Finally, they were asked questions about their symptoms and the management of these. See Appendix 1 for the UK version of the survey. This version was revised and updated after being distributed in the UK, before being translated and distributed in Germany and Italy.

### Survey sampling

Participation in the survey was open to all patients with ataxia (or carers of patients with ataxia, who could act as proxy respondents), who were aged 16 years old or more. The ataxia patient associations in each country (Ataxia UK in the UK; Deutsche Heredo Ataxie Gesellschaft (DHAG) and Ataxie Forderverein e.V. in Germany; Associazione Italiana per la lotta alle Sindromi Atassiche (AISA) in Italy) publicised the surveys to potential participants via newsletters, social media, website and events. In the UK the survey was mainly distributed online via Ataxia UK’s mailing list, website, magazine and social media channels. In Germany and Italy the survey was publicised via the patients associations and also via clinicians working at SACs. In the UK the survey was submitted for ethical approval via the Integrated Research Application System (IRAS; reference 252,966) and received approval by the Cambridge Research Ethics Committee (REC; reference 19/EE/0030). As part of this process all materials related to the survey, including the patient information sheets and final questionnaire, were validated by a clinical expert and an informal review panel including ataxia patients, nominated by Ataxia UK. For the two other countries, ethical approval for an anonymised survey was not needed. The survey was ‘live’ from March to May 2019 in the UK, from February to October 2020 in Germany, and from May to September 2021 in Italy; note the different time periods.

### Data analysis

Responses were removed for all respondents who did not provide informed consent or who did not provide positive responses to the three screening questions contained within survey (questions 3, 4 and 5; see Appendix 1). Where the respondent gave clear contradictory responses, the responses to those questions were removed from the analysis. Incomplete surveys were not removed from the analysis, as respondents chose to answer some questions and to skip others. The database used for cleaning and analysis was anonymised.

Participants were asked to record the number of health care contacts they had received in the preceding 6 (in the case of the UK) or 12 (in Germany and Italy) months specifically to manage their ataxia. Participants were asked to record the number of health care contacts with the general practitioner (GP), hospital outpatient clinic visits with a neurologist (not at a SAC), SAC visits, hospital inpatient stays, accident and emergency department visits, physiotherapy appointments, speech and language therapy appointments, occupational therapy appointments, and other consultant specialist visits (e.g. an ophthalmologist, an ear, nose and throat specialist, urologist, gastroenterologist and others). Figures for the UK were multiplied by two to give 12-month estimates, commensurate with the other two counties [11]. The wording in the UK version of the questionnaire could have been misunderstood, as respondents were asked to report the number visits to the specialist ataxia centre, and this may have been interpreted by respondents to mean the number of visits to see a specialist about their ataxia at the hospital where the SAC was, irrespective of the type of specialist seen or whether or not the visit was at a SAC. We know that according to routine practice in the UK people with ataxia who attended a SAC are invited to visit the centre once per year. Therefore, all UK respondents who reported attending the SAC were calculated to have one visit to the SAC each year and any additional visits that were reported were included in the economic analysis as other consultant specialist. Unit costs for each type of contact were obtained from local providers or published sources [12, 13]. All costs were calculated in 2021 €; 2021 unit costs for the UK were reported in UK£ and converted into € using GDP Purchasing Power Parities; unit costs for Germany and Italy were reported in €; prices were converted into 2021 values using consumer price indices for each country. Given the small numbers of respondents, and the variation in the numbers of respondents to each resource use question, we multiplied the unit cost by the mean volume of resource use for each type of resource use and summed these across all types of resource use to calculate mean annual treatment costs per patient per annum in each of the three countries.

Participants were also asked whether they were currently attending or had ever attended a SAC. Participants who reported they had never been to a SAC were grouped in the “non-SAC” group; those who reported they were currently attending a SAC for some or all of their treatment were grouped in the “SAC” group. A third group comprising people who used to go to a SAC for some or all of their treatment but no longer attend were not included in the analysis; the number of respondents in this group was small, and it was unclear whether their resource use and costs would be affected by their previous contact with the SAC.

We compared average resource use for each contact type, and costs, over a 12-month period, stratifying patients by whether they were currently attending a SAC or had never attended a SAC. We tested for significant differences in mean resource use and costs between the SAC/non-SAC groups in each country using unadjusted and adjusted ordinary least squares regression analysis, the latter controlling for age, sex, number of symptoms experienced as a result of ataxia, and whether or not the patient had comorbidities. We also tested for significant differences in mean contacts and costs between countries separately for non-SAC and SAC groups using adjusted ordinary least squares regression analysis, controlling for age, sex, number of symptoms experienced as a result of ataxia, and whether or not the patient had comorbidities.

## Results

After cleaning the data, we had 277 respondents from the UK, 101 from Germany and 174 from Italy. Over three-quarters of all respondents in each country were patients (as opposed to parents/carers responding on behalf of the patient; Table 1). There were differences in the characteristics of the respondents between countries. The modal age category in each country was 60–79 years in the UK, and 30–59 years in Italy and Germany. The proportion of respondents who were female was 53%, 54% and 48%, respectively. In the UK the most prevalent ataxia diagnosis in the sample was idiopathic cerebellar ataxia (43%); in Italy it was Friedreich’s ataxia (35%); in Germany it was inherited cerebellar ataxia (55%). The percentage of sample respondents in each country who reported they were currently attending a SAC was 29%, 59% and 57%, for UK, Italy and Germany, respectively. After removing participants who said they used to go to a SAC but were not currently going, and/or who answered none of the resource use questions, we had responses from 181 patients from the UK, 96 patients from Italy and 43 patients from Germany; this was our final sample, and comprised those who said they were either currently attending a SAC or had never attended a SAC and provided resource use data.

**Table 1:** Survey sample characteristics

Characteristic	UK	Italy	Germany
**Respondent type**			
Patient	234 (85.7%)	131 (75.7%)	90 (89.1%)
On behalf of the patient	39 (14.3%)	42 (24.3%)	11 (10.9%)
**Age (years)**			
16–29	12 (4.4%)	22 (12.72%)	12 (11.9%)
30–59	106 (39.3%)	115 (66.5%)	60 (59.4%)
60–79	140 (51.9%)	35 (20.2%)	29 (28.7%)
80+	12 (4.4%)	1 (0.6%)	0 (0%)
**Sex**			
Female	142 (52.6%)	93 (53.8%)	48 (47.5%)
Male	128 (47.4%)	80 (46.2%)	53 (52.5%)
**Diagnosis**			
FRDA	27 (10.1%)	56 (35.2%)	14 (14.9%)
Inherited CA	78 (29.2%)	42 (26.4%)	52 (55.3%)
Idiopathic CA	114 (42.7%)	19 (12%)	
Other types	38 (14.2%)	27 (17%)	15 (16%)
Not known	10 (3.8%)	15 (9.4%)	13 (13.8%)
**Attendance at SAC**			
Never been	128 (51.6%)	27 (19.4%)	23 (23.4%)
Currently going	72 (29%)	82 (59%)	48 (57.1%)
Used to go	48 (19.4%)	30 (21.6%)	13 (15.5%)

CA, cerebellar ataxia; FRDA, Friedreich’s ataxia; SAC, specialist ataxia centre

After data cleaning, there were 277 respondents to the survey in the UK, 101 in Germany, and 174 in Italy; not all respondents answered every question

Figures are numbers in each category (%)

In the UK the most common contacts for SAC patients were physiotherapy visits (mean 3.1 visits per patient per year) followed by general practitioner visits and other visits (1.9) (Table 2). In Italy the most common contacts were physiotherapy visits (14.5) and speech and language therapy visits (6.4). In Germany the most common contacts were physiotherapy visits (27.9), speech and language therapy visits (11.5) and occupational health therapy visits (10.4). In every country the differences in the numbers of contacts for the other types of health service use between the SAC and non-SAC groups were mostly non-significant. The exception to this in the UK was the number of other visits to specialists (other than neurologists): there were higher mean contacts for patients in the SAC group compared to the non-SAC group (*P* < 0.01). In Italy, neurology outpatient visits were different between the SAC and non-SAC groups, with higher mean contacts in the non-SAC group (*P* = 0.02). In Germany, patients in the SAC group had higher mean contacts for speech and language therapy visits (*P* = 0.02). For the non-SAC group there were differences between countries in terms of general practitioner visits (the highest number of visits were in Germany, then the UK, then Italy, *P* < 0.01), physiotherapy visits (Germany, Italy, then the UK), speech and language therapy visits (Germany, Italy, then the UK, *P* < 0.01) and occupational health therapy visits (Germany, Italy, then the UK, *P* < 0.01). For the SAC group there were significant differences between countries in terms of specialist centre visits (the highest number of visits were in Italy, then Germany, then the UK, *P* = 0.01), General Practitioner visits (Germany, the UK, then Italy, *P* < 0.01), neurologist outpatient visits (Germany, the UK, then Italy, *P* < 0.01), physiotherapy visits (Germany, Italy, then the UK), speech and language therapy visits (Germany, Italy, then the UK, *P* < 0.01) and occupational health therapy visits (Germany, Italy, then the UK, *P* < 0.01).

**Table 2:** Health care contacts over a one-year period for non-SAC and SAC patients in three European countries

	Patients who reported never attending a SAC				Patients who reported attending a SAC currently					
Health care contacts	N	Mean	Std. Dev.	Median	N	Mean	Std. Dev.	Median	***P***-value†	***P***-value‡
**United Kingdom**										
Specialist centre visits	109	0	0	0	72	1	0	1	< 0.01	< 0.01
General Practitioner visits	108	2.3	3.5	0	58	1.9	3.3	0	0.40	0.51
Neurologist outpatient visits	115	1.5	2.9	0	59	1.3	2.6	0	0.54	0.24
Inpatient stays	110	0.3	1.0	0	58	0.1	0.4	0	0.20	0.17
Accident & Emergency visits	112	0.3	1.0	0	60	0.3	1.0	0	0.65	0.82
Physiotherapy visits	109	1.6	3.2	0	64	3.1	4.5	0	0.01	0.11
Speech and Language therapy visits	113	0.7	1.8	0	60	0.3	0.7	0	0.06	0.23
Occupational Health Therapy visits	113	1.3	3.1	0	63	1.5	3.0	0	0.81	0.98
Other consultant specialist visits	109	0.7	2.1	0	59	3.0	3.3	1	< 0.01	< 0.01
**Italy**										
Specialist centre visits	22	0	0	0	74	1.4	0.7	1	< 0.01	< 0.01
General Practitioner visits	19	1.3	2.6	0	54	0.7	1.3	0	0.20	0.39
Neurologist outpatient visits	20	2.7	5.0	1.5	55	0.4	0.7	0	< 0.01	0.02
Inpatient stays	19	0.3	0.6	0	57	0.3	0.6	0	0.91	0.51
Accident & Emergency visits	18	0.3	0.8	0	57	0.1	0.4	0	0.30	0.17
Physiotherapy visits	21	19.0	19.6	10	63	14.5	19.8	2	0.37	0.40
Speech and Language therapy visits	19	7.8	11.6	1	61	6.4	12.7	1	0.67	0.78
Occupational Health Therapy visits	18	1.5	2.7	0	50	1.3	4.1	0	0.88	0.95
Other consultant specialist	18	1.6	4.0	0	59	1.9	5.5	0	0.80	0.28
**Germany**										
Specialist centre visits	10	0.0	0.0	0	33	1.3	0.9	1	< 0.01	0.01
General Practitioner visits	8	5.5	10.1	2	34	4.9	5.2	3.5	0.80	0.23
Neurologist outpatient visits	11	3.7	3.1	3	30	2.5	2.9	2	0.23	0.47
Inpatient stays	10	0.0	0.0	0	31	0.3	0.7	0	0.24	0.24
Accident & Emergency visits	9	0.1	0.3	0	31	0.6	2.7	0	0.59	0.92
Physiotherapy visits	11	33.7	22.8	50	28	27.9	23.7	40	0.49	0.92
Speech and Language therapy visits	10	8.9	18.5	0	33	11.5	17.2	1	0.69	0.02
Occupational Health Therapy visits	10	7.9	17.2	0	30	10.4	17.7	0	0.70	0.41
Other consultant specialist visits	9	1.6	2.3	1	28	1.6	2.0	1	0.95	0.54
**P-value**§										
Specialist centre visits	-				0.01					
General Practitioner visits	< 0.01				< 0.01					
Neurologist outpatient visits	0.10				< 0.01					
Inpatient stays	0.92				0.35					
Accident & Emergency visits	0.53				0.18					
Physiotherapy visits	< 0.01				< 0.01					
Speech and Language therapy visits	< 0.01				< 0.01					
Occupational Health Therapy visits	< 0.01				< 0.01					
Other consultant specialist visits	0.16				0.30					

† Test for significant differences in mean contacts between non-SAC and SAC groups (unadjusted)

‡ Test for significant differences in mean contacts between non-SAC and SAC groups (adjusted for age, sex, number of symptoms and number of comorbidities)

§ Test for significant differences in mean contacts between countries separately for non-SAC and SAC groups (adjusted for age, sex, number of symptoms and number of comorbidities)

SAC, specialist ataxia centre; N, number of participants who responded to that question

There were differences in unit costs between countries for every type of health care contact (Table 3). In the UK the mean total cost per patient over a one-year period was €2209 for non-SAC patients and €1813 for SAC patients (*P* = 0.59). In Italy these figures were €2126 and €1971, respectively (*P* = 0.84). In Germany they were €2431 and €4087 (*P* = 0.19), respectively. Within every country the difference in mean costs per patient between the non-SAC and SAC groups was not statistically significantly different from zero (Table 3). In every country the health care contact that contributed most to the total cost was inpatient stays (Table 3); while the mean number of inpatient stays per person was relatively low (Table 2), the relatively high unit cost per inpatient stay meant that this type of contact contributed substantially to overall cost. For the non-SAC group there were no significant differences in mean costs between countries (*P* = 0.95), though there was a trend for higher costs in Germany. For the SAC group there were significant differences in mean costs between countries, with mean costs per patient highest in Germany with similar lower costs in Italy and the UK (*P* < 0.01; Table 3).

**Table 3:** Costs of health care contacts over a one-year period for non-SAC and SAC patients in three European countries

	United Kingdom			Italy			Germany		
Health care contacts	Unit cost	Cost non-SAC	Cost SAC	Unit cost	Cost non-SAC	Cost SAC	Unit cost	Cost non-SAC	Cost SAC
Specialist centre visits	197	0	197	25	0	36	170	0	221
General Practitioner visits	42	98	81	25	32	17	29	161	144
Neurologist outpatient visits	197	296	256	25	69	10	65	242	163
Inpatient stays	4280	1284	428	4734	1420	1420	4950	0	1485
Accident & Emergency visits	196	59	59	24	7	2	144	14	86
Physiotherapy visits	65	104	201	20	374	285	38	1274	1055
Speech and Language therapy visits	185	130	56	20	154	126	38	337	435
Occupational Health Therapy visits	120	156	181	20	30	26	38	299	393
Other consultant specialist visits	119	83	355	25	41	48	65	104	104
Total		2209	1813		2126	1971		2431	4087
**P-value**†			0.55			0.68			0.26
**P-value**‡			0.59			0.84			0.19

† Test for significant differences in mean costs between non-SAC and SAC groups (unadjusted)

‡ Test for significant differences in mean costs between non-SAC and SAC groups (adjusted for age, sex, number of symptoms and number of comorbidities)

SAC, specialist ataxia centre

All numbers are 2021 €

The numbers in the Cost non-SAC and Cost SAC columns are the unit costs for each type of health care contact multiplied by the number of those contacts in Table 2. We tested for significant differences in mean costs between countries separately for non-SAC and SAC groups (adjusted for age, sex, number of symptoms and number of comorbidities). For the non-SAC group there were no significant differences in mean costs between countries (*P*-value = 0.95). For the SAC group there were significant differences in mean costs between countries (*P*-value < 0.01)

## Discussion

### Summary of findings

The ataxias are complex rare neurological disorders with no approved therapies, no disease modifying treatments, and despite the presence of guidelines for the management of ataxia, it is not known if they are implemented in practice by health care professionals. Our findings show that a range of health care professionals are involved in the management of ataxia in Europe.

The resource use data presented in this study show that within countries there is little variation in resource use, depending on whether or not patients attended a SAC; there is however variation between countries in some types of health care contact, most notably in terms of physiotherapy visits, speech and language therapy visits, and occupation health therapy visits, where for each of these the highest number of visits were in Germany, then Italy, then the UK.

In terms of costs of care, in the UK and in Italy mean costs were slightly numerically higher in the non-SAC groups compared with the SAC groups; however the differences per patient over a one-year period were small (€396 in the UK, €155 in Italy) and the differences were not statistically significant. In Germany, mean costs were numerically higher in the SAC group (a difference of €1656 per patient), and the differences were also not statistically significant. In terms of between-country comparisons, for the non-SAC group there were no significant differences in mean costs between countries, though there was a trend for higher costs in Germany. For the SAC group there were significant differences in mean costs between countries, with mean costs per patient highest in Germany and similar lower costs in Italy and the UK. The higher numerical value of costs in Germany compared with the UK and Italy reflects differences in overall health expenditure per capita between the three countries;^13^ in Germany mean overall spending on health per capita is the fourth highest across all OECD countries (US$6518, including both government/compulsory spending and voluntary/out-of-pocket spending), whereas in the UK and Italy health spending per capita is around the OECD average (US$4087) [14].

### Strengths and weaknesses

Strengths of this study were that it included ataxia patients in three different countries, and included detailed resource use data collected from surveys with a large combined sample overall of 552 respondents. Limitations are the relatively small numbers of patients from each country, especially when disaggregated by whether or not they visited a SAC; this means that the findings may not fully reflect the experiences of the ataxia community, though the relatively small numbers reflect the fact that ataxia is a rare condition. We also point out that there were differences in the characteristics of the respondents between countries, and while we controlled for age, sex, number of symptoms experienced as a result of ataxia, and whether or not the patient had comorbidities, this variation in characteristics might affect the comparability of the findings between countries. In addition, the time periods during which data collection occurred for Germany and Italy may have been affected by the COVID-19 pandemic, which may have affected access to health care services and resource use. In both countries, while the number of face-to-face contacts may have fallen, since contacts with people affected by neurodegenerative disorders will still occur these are likely to have been replaced by virtual contacts, thereby not altering the total numbers of contacts. Cost comparisons between the three countries are difficult given that these costs may be affected by differences in resource use and unit costs for different types of health care contact, and these will depend at least in part on the different prevailing health care financing systems. We also note that resource use data for Italy and Germany were collected for the preceding 12 months, whereas for the UK they were only collected for the previous 6 months. To make the figures more comparable we multiplied the UK figures by two [11]. We acknowledge this might not completely reflect 12 months resource use in the UK, however we also compared our survey data with data from clinical practice in the two UK specialist ataxia centres and the numbers were broadly consistent. We also note that the use of retrospective resource use data may be prone to recall problems. The focus of this study was health service contacts and costs, and we have underestimated the costs associated with ataxia because we did not include non-health service costs (e.g., out-of-pocket expenses and costs of time off work); as shown by Giunti et al. [15], these may be substantial for people affected by ataxia.

### Further research

Further research would be useful to understand the differences in resource use and costs between SAC and non-SAC groups within countries, and the differences in resource use and costs between countries. Research to relate the costs associated with attending a SAC with the impact on health outcomes would also be useful, to understand how best to allocate resources for treating patients with ataxia efficiently. Further research would also be useful to explore the impact of being treated at a SAC on health-related quality of life and other costs associated with living with ataxias, e.g., out-of-pocket expenses and time off work.

### Electronic supplementary material

Below is the link to the electronic supplementary material.

Supplementary Material 1: Appendix 1. Patient survey distributed in the UK

Supplementary Material 2: Dataset of participants survey for the three countries

## Data Availability

The data that support the findings of this study are available as an excel file added in the supplementary material at the end of the manuscript. In order to conceal anonymity of the participants, we have removed their location.
